# Impact of improved attenuation correction on 18F-FDG PET/MR hybrid imaging of the heart

**DOI:** 10.1371/journal.pone.0214095

**Published:** 2019-03-25

**Authors:** Maike E. Lindemann, Felix Nensa, Harald H. Quick

**Affiliations:** 1 High-Field and Hybrid MR Imaging, University Hospital Essen, University Duisburg-Essen, Essen, Germany; 2 Department of Diagnostic and Interventional Radiology and Neuroradiology, University Hospital Essen, University of Duisburg-Essen, Essen, Germany; 3 Erwin L. Hahn Institute for Magnetic Resonance Imaging, University Duisburg-Essen, Essen, Germany; Ente Ospedaliero Cantonale, SWITZERLAND

## Abstract

**Purpose:**

The aim of this study was to evaluate and quantify the effect of improved attenuation correction (AC) including bone segmentation and truncation correction on 18F-Fluordesoxyglucose cardiac positron emission tomography/magnetic resonance (PET/MR) imaging.

**Methods:**

PET data of 32 cardiac PET/MR datasets were reconstructed with three different AC-maps (1. Dixon-VIBE only, 2. HUGE truncation correction and bone segmentation, 3. MLAA). The Dixon-VIBE AC-maps served as reference of reconstructed PET data. 17-segment short-axis polar plots of the left ventricle were analyzed regarding the impact of each of the three AC methods on PET quantification in cardiac PET/MR imaging. Non-AC PET images were segmented to specify the amount of truncation in the Dixon-VIBE AC-map serving as a reference. All AC-maps were evaluated for artifacts.

**Results:**

Using HUGE + bone AC results in a homogeneous gain of ca. 6% and for MLAA 8% of PET signal distribution across the myocardium of the left ventricle over all patients compared to Dixon-VIBE AC only. Maximal relative differences up to 18% were observed in segment 17 (apex). The body volume truncation of -12.7 ± 7.1% compared to the segmented non-AC PET images using the Dixon-VIBE AC method was reduced to -1.9 ± 3.9% using HUGE and 7.8 ± 8.3% using MLAA. In each patient, a systematic overestimation in AC-map volume was observed when applying MLAA. Quantitative impact of artifacts showed regional differences up to 6% within single segments of the myocardium.

**Conclusions:**

Improved AC including bone segmentation and truncation correction in cardiac PET/MR imaging is important to ensure best possible diagnostic quality and PET quantification. The results exhibited an overestimation of AC-map volume using MLAA, while HUGE resulted in a more realistic body contouring. Incorporation of bone segmentation into the Dixon-VIBE AC-map resulted in homogeneous gain in PET signal distribution across the myocardium. The majority of observed AC-map artifacts did not significantly affect the quantitative assessment of the myocardium.

## Introduction

The integration of positron emission tomography (PET) and magnetic resonance (MR) imaging into one PET/MR hybrid system [1] [2] has shown great potential in various cardiovascular applications with regard to evaluation of cardiac viability and function and diagnosis of tumor or inflammation [3] [4] [5] [6] [7].

Technical challenges remain applying PET/MR to the cardiovascular system [8] [9] [10] [11] [12]. Attenuation correction (AC) of PET data is an essential step in obtaining accurate and quantitative PET images [13] [14]. State of the art in PET/MR hybrid imaging is a segmentation approach based on a Dixon-VIBE MR sequence, which divides the MR image into four tissue classes (background air, lung, fat and soft tissue) and assigns predefined linear attenuation coefficients (LACs) to the segmented tissue regions [15].

The established MR-based methods of creating AC-maps have certain limitations compared to computer tomography (CT) based AC [16], e.g. the substitution of bone as soft tissue, which may lead to a systematical underestimation in PET signal [17]. Another constraint of MR-based AC is the limitation of the MR field-of-view (FOV) to a diameter of about 50 cm due to hardware restrictions such as B_0_ inhomogeneities and gradient nonlinearities. Hence, truncation artifacts may occur at off-center positions and lead to bias in the entire AC-map, and thus in PET activity quantification [18]. Truncation artifacts frequently occur along the arms that rest beside the patient during PET/MR examinations [18]. Regarding the lack of bone attenuation correction, a prototype model-based approach including the LAC of the major bones in the standard Dixon-VIBE AC-map was recently tested. The bias in PET quantification due to MR-based AC was reduced significantly [19] [20]. Regarding the FOV limitations in AC-maps, several methods have been explored to complement truncation artefacts in the MR-based AC-map. One approach is based on joint estimation of emission and transmission data using the maximum likelihood estimation of activity and attenuation (MLAA) image reconstruction method, a PET-based method to calculate a truncation corrected AC-map [21] [22]. A fully MR-based approach for truncation correction is HUGE (B_0_ Homogenization Using Gradient Enhancement), which optimizes the readout gradient to locally compensate the B_0_ inhomogeneities, and thus the truncations [23] [24] [25].

While in another recent study the quantitative impact of artifacts in PET/MR AC on cardiac imaging has been thoroughly investigated [9], in this present study we investigate the quantitative impact of the most current improvements of MR-based attenuation correction, namely HUGE truncation correction [25] and bone segmentation [19] on 18F-FDG PET quantification in cardiac PET/MR. The established methods Dixon-based AC and MLAA PET-based truncation correction served as intraindividual reference in this study. The AC methods were applied in 32 cardiac patient datasets. Multiple reconstructions of the PET data with three different AC-maps for each patient were analyzed regarding the impact of each approach on PET quantification in cardiac PET/MR imaging.

## Material and methods

### Patient population

In this study, 32 patients underwent a cardiac 18F-FDG PET/MR examination. The patient population consists of 8 female and 24 male patients (mean age 56.8 ± 13.7 years, mean body-mass-index (BMI) 26.9 ± 4.6 kg/m^2^), who were administered an average radiotracer dosage of 184.7 ± 63.4 MBq. The PET/MR measurement started 72.2 ± 21.1 minutes post injection. Patients were referred to cardiac PET/MR because of suspected cardiac sarcoidosis (n = 11), myocarditis (n = 9) endocarditis (n = 6), cardiac tumors (n = 3), and myocardial ischemia (n = 3). 29 patients fasted before PET/MR examination to suppress FDG enhancement in the normal myocardium. 3 patients with suspected myocardial ischemia got glucose before PET/MR acquisition to achieve myocardial FDG enhancement. The study was conducted in conformance with the Declaration of Helsinki and approved by the Ethics Commission of the Medical Faculty of the University Duisburg-Essen (study number 11–4822-BO), and all patients provided written informed consent before examination.

### Attenuation correction

The attenuation due to rigid hardware components such as the patient table, the spine array radiofrequency (RF) coil and the head/neck RF coil is corrected using CT-based AC templates of the respective RF coil, as implemented by the manufacturer of the PET/MR system. The AC of hardware components is automatically performed by the system based on an established method [26]. AC of the flexible body coils in PET/MR hybrid imaging is not straightforward since the position and the geometry of the flexible RF coils are not known during the examination. Thus, flexible RF coils are designed such that they provide only low attenuation of photons. Hence, the attenuation bias due to flexible RF coils are in the range of a few percent only [27].

State of the art in PET/MR hybrid imaging is a segmentation approach based on a Dixon-VIBE MR sequence, which divides the MR image into four tissue classes and assigns predefined linear attenuation coefficients to the segmented tissue regions (background air 0.0 cm^-1^, lung 0.0224 cm^-1^, fat 0.0854 cm^-1^ and soft tissue 0.1 cm^-1^) [15]. Sequence parameters of the Dixon-VIBE technique used in this study are: image matrix 192 x 126 x 128, resolution 2.6 x 2.6 x 3.1 mm^3^, TE1 1.23 ms, TE2 2.46 ms, TR 3.6 ms, FA 10°, TA 19 s per bed. Due to hardware restrictions in MR imaging causing magnetic field inhomogeneities and gradient non-linearities, the MR FOV is typically limited to a spherical volume with a diameter of approximately 50 cm. Therefore, the MR-based AC-map may be truncated along the patients’ arms as their lateral position parallel to the patient body may exceed the dimensions of the MR FOV.

MLAA is a PET-based approach to correct the truncations in the AC-map by predicting the missing body contour from PET emission data. This method is based on joint estimation of emission and transmission data using the maximum likelihood estimation of activity and attenuation (MLAA) image reconstruction method. It assumes radioactive tracer uptake in the truncated parts of the arm to derive accurate body contours from PET data. In this study, the activity and attenuation maps were reconstructed with 1 iteration and 1 subset of the OSEM and 20 iterations and 9 subsets of MLAA algorithm. Matrix size of the completed AC-map was 344 x 344 x 127 with a resolution of 2.09 x 2.09 x 2.03 mm^3^. The truncation corrected MLAA AC-map is based on the Dixon-VIBE AC-map complemented with MLAA information from the arms [21] [22].

A purely MR-based approach for MR FOV extension is HUGE (B_0_ Homogenization Using Gradient Enhancement). This method corrects truncations in the AC-map by using system specific field plots to calculate space-dependent optimal readout gradients. These optimized readout gradients locally compensate B_0_ inhomogeneities, and thus achieve a reduction in truncations outside the regular FOV. The HUGE method used in this study was applied as follows. Patients’ arms for left and right side were measured separately with a modified HASTE (Half Fourier-Acquired Single shot Turbo spin Echo) sequence with a slice thickness of 8 mm in z-direction. Protocol parameters for each side were: image matrix 128 x 128, resolution 2.3 x 2.3 mm^2^, TE 34 ms, TR 1610 ms, FA 180°. HUGE is combined with continuous table motion, such that HUGE data is always acquired in the most homogeneous region of the magnet iso-center where also the imaging gradients provide best linearity. The table speed was set to 27.4 mm/s. Thus, the overall data acquisition time for HUGE to cover the patient’s arms with a standardized table distance in the range of 500–1300 mm (two directions, one for each arm) was between 40 and 90 s. The truncation corrected HUGE AC-map is based on the Dixon-VIBE AC-map complemented with HUGE information from the arms [23] [24].

The substitution of bone as soft tissue in the AC-map may lead to a systematical underestimation of PET signal in PET/MR imaging [17]. To provide AC for bone, a model-based bone segmentation approach was introduced to complement the Dixon-based soft tissue AC-map [20] [19]. This method includes a set of pre-aligned MR image and bone mask pairs for each major body bone (skull, spine, pelvis and upper femur) individually. The bone model as described by Paulus et al. was generated from a data base of more than 200 Dixon-VIBE images and bone mask pairs. For each major bone, the MR image from the model is registered to the MR image of each patient. Bone masks were chosen from the data base best depicting the shape and density of the average of the pool. The model is registered to the individual Dixon-VIBE images of each patient. After registration continuous LACs (0.1 cm^−1^ up to 0.2485 cm^−1^) for bone are then added to the Dixon-VIBE AC-map [20] [19]. This bone model was also implemented in the AC-maps of the present study to correct for attenuation due to major bones.

### Image acquisition and reconstruction

**Fig 1:**
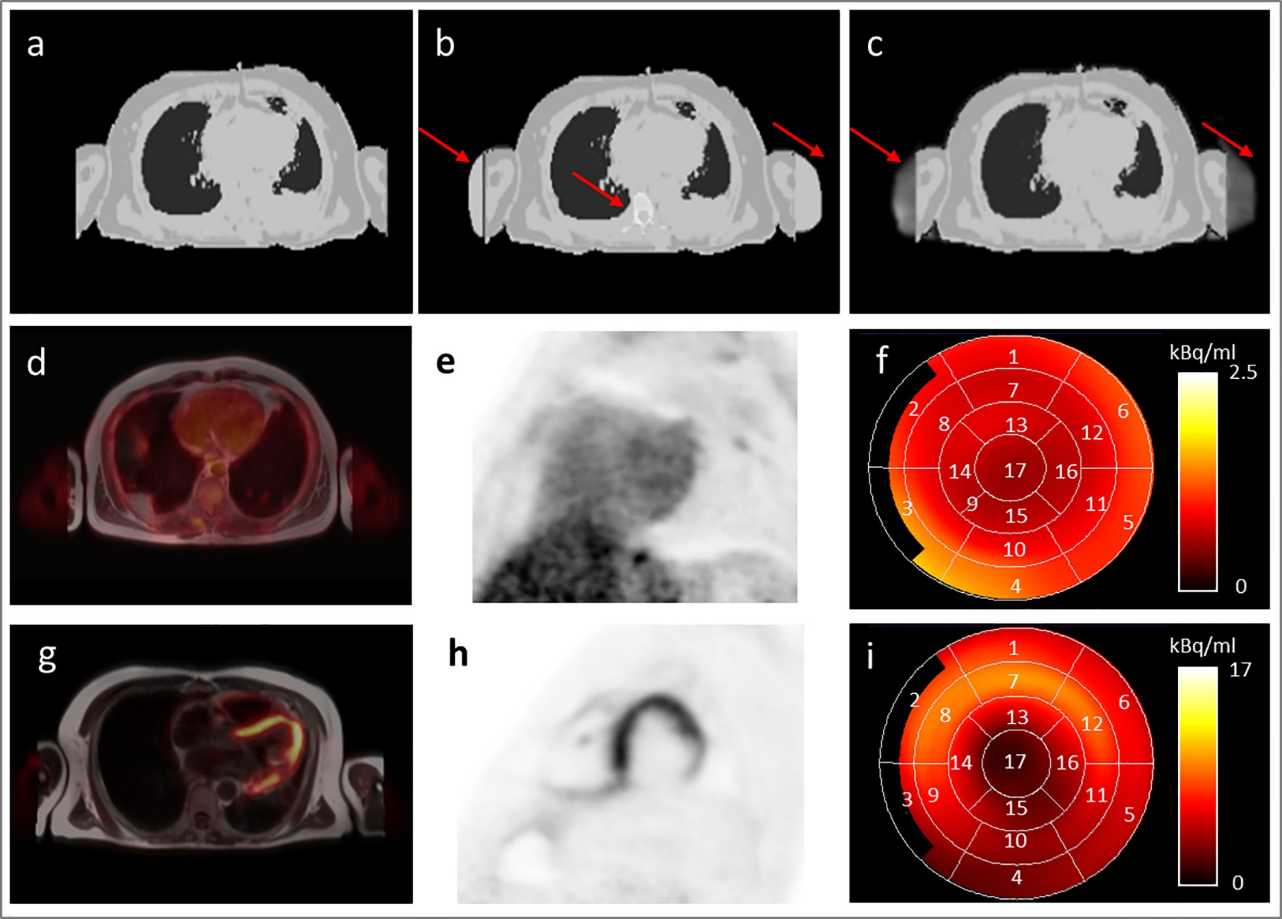
Overview of AC-maps, cardiac PET data and polar plots. Attenuation correction (AC) maps based on Dixon-VIBE sequence (a), the extended Dixon-VIBE sequence additionally using HUGE truncation correction and bone information (b), and the extended Dixon-VIBE sequence additionally using PET-based MLAA information (c). Red arrows depict differences between Dixon-VIBE only AC and improved AC-maps using HUGE, bone segmentation, and MLAA, respectively (b, c). Patient example studied in the fasted state (d-f) and patient example studied with a viability protocol (g-i) are shown. PET/MR fusion image (d, g) and PET short axis view (e, h) of the heart. A 17-segment polar plot (f, i) of the short-axis of the left ventricle was generated for each patient and each PET data reconstruction to evaluate the quantitative effect of improved AC on cardiac PET imaging. Numbers 1–17 are assigned to the 17 individual cardiac segments according to the American Heart Association standard for cardiac polar plots.

All PET/MR measurements were performed on an integrated 3-Tesla whole-body PET/MR system (Biograph mMR, Siemens Healthcare GmbH, Erlangen, Germany). For attenuation correction, the PET/MR acquisition protocol consists of a standard Dixon-VIBE sequence and the HUGE sequence for FOV extension in x-y-direction. To validate the impact of truncation correction and bone segmentation on cardiac PET/MR imaging, all PET data were reconstructed three times with different AC-maps: 1. Dixon-VIBE only, 2. HUGE truncation correction and bone segmentation, and 3. MLAA truncation correction (Fig 1). The Dixon-VIBE AC-map serves as the reference for all three comparisons of reconstructed PET data. The second AC-map consists of Dixon-VIBE supplemented with MR-based HUGE information from extended FOV and segmented bone information. The third MLAA AC-map composites of Dixon-VIBE AC-map extended with the PET-based MLAA method (no bone segmentation). All PET reconstructions were performed using the OP-OSEM algorithm with 3 iterations, 21 subsets and 4 mm Gaussian filter. AC-maps with artifacts were also considered in the patient population.

### Image analysis

The truncation of body volume in the Dixon-VIBE AC-map was quantified to use this as a basis for assessment of performance of the two truncation correction methods, HUGE and MLAA. For this we use body-contour delineation to specify the amount of body volume truncation in the standard Dixon-VIBE AC-map and based on this HUGE and MLAA could be quantitatively evaluated knowing the true truncated volume for each patient data set. For contour delineation non-attenuation corrected (NAC) PET data was used [24]. To evaluate the quantitative effect of different AC-maps on cardiac PET data polar plots of the left ventricles in short axis orientation were generated using Carimas v2.9 [28] according to the 17-segment model of the American Heart Association (AHA) [29] (Fig 1). Since the majority of the patients had a dietary suppression of myocardial glucose uptake, the left ventricular myocardium was segmented from short-axis MR images to ensure that the uptake is always measured at identical location within the thickness of the end-systolic myocardium for all reconstructions. To compare the polar plots from different reconstructions for each patient, it was refrained from normalizing the polar plots. To normalize the uptake, regional uptake in a specific location is defined as a reference. In PET data corrected with different AC-maps this location might change, and therefore might lead to unexpected results. In each segment of the polar plots relative differences between the standard Dixon-VIBE AC and the improved AC using HUGE truncation correction and bone segmentation or MLAA were calculated. Bland-Altman plots were generated to compare the two improved AC methods HUGE/bone and MLAA AC-map in AC-map volume and activity in the left ventricle. The difference in volume between standard AC (Dixon-VIBE) and improved AC (HUGE or MLAA) refer to the added volume along the arms due to the FOV extension. Significance for differences in volume and signal were calculated. P-values < 0.05 were considered to be statistically significant.

## Results

**Fig 2:**
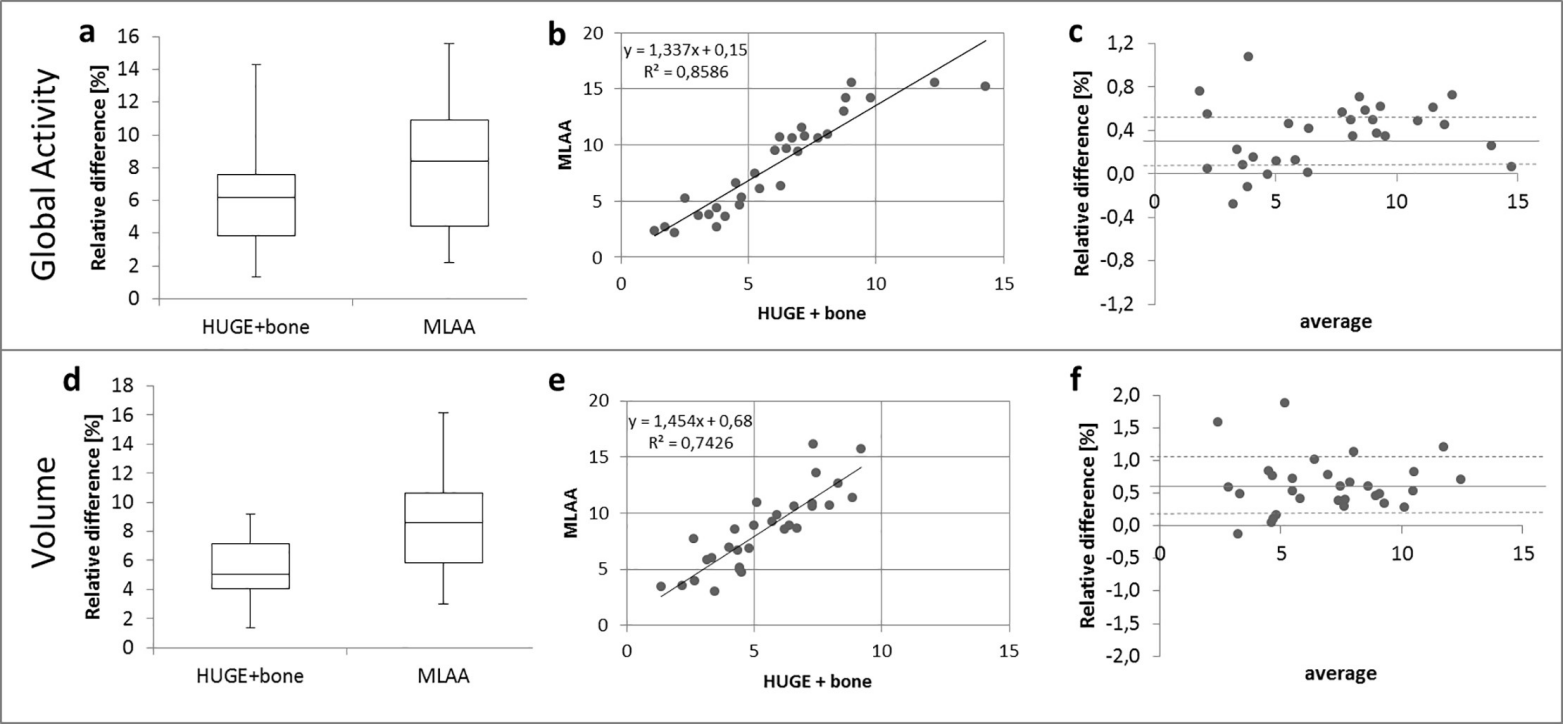
Statistical analysis of quantitative impact of improved AC on AC-map volume and PET signal. Statistical analysis of quantitative impact (global activity) of HUGE field-of-view extension, bone segmentation and MLAA correction on global PET signal over all segments of the left ventricle (a-c). Increase of AC-map volume (d-f) relative to AC-map volume as provided by standard Dixon-VIBE AC imaging. Boxplots (a, d) show the statistical distribution in relative gain of activity and volume from applying improved AC. Correlation graphs (b, e) and Bland-Altman plots (c, e) depict the comparison between HUGE + bone and MLAA in contrast to standard Dixon-VIBE AC serving as reference. In the correlation graphs (b, e) the linear equation with the coefficient of determination (R^2^) is given.

Fig 2 shows the statistical analysis of the impact of truncation correction and bone segmentation on global PET signal over all segments of the left ventricle and AC-map volume relative to standard Dixon-VIBE AC imaging for all patient data sets. Considering all 32 patients, the average AC-map volume increase using the HUGE method for truncation correction is 5.4 ± 2.0% and using the MLAA approach is 8.5 ± 3.4% when compared to the standard Dixon-VIBE AC-map (Fig 2D). The total range of volume increase across the patient collective is 1.4% up to 9.2% for HUGE and 3.0% - 16.2% for MLAA. The differences in AC-map volume are statistically significant (p < 0.05) for both FOV extension methods HUGE and MLAA. The average gain over all patients and segments in PET activity in the left ventricle for HUGE + bone is 6.1 ± 3.0% and for MLAA 8.3 ± 4.3% compared to Dixon-VIBE imaging only (Fig 2A). The total range of activity increase, considering all 32 patients individually, is 0.4% up to 18.8% for HUGE and 0.9% - 19.8% for MLAA. The differences in activity are statistically significant (p < 0.05) for both FOV extension methods HUGE and MLAA. The correlation of AC-map volume and PET activity in the left ventricle between HUGE + bone and MLAA depicts, that nearly all patients gain more AC-map volume with MLAA, however, in corresponding activity results the impact of both methods is comparable (Fig 2B and 2E). Bland-Altman plots show the relative overestimation in AC-map volume due to MLAA result in systematically higher count statistic (Fig 2C and 2F).

**Fig 3:**
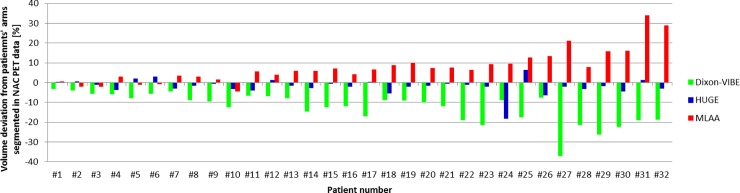
Percentage volume deviations of the patients’ arms compared to the segmented NAC PET images. Percentage volume deviations of the patients’ arms in the Dixon-VIBE, HUGE and MLAA attenuation correction maps compared to the patient’s arms segmented in the non-attenuation corrected (NAC) PET data. All 32 patients are sorted by increasing body mass index (BMI) from left to right. Note the tendency of increased body volume truncation percentage for patients with higher BMI.

Fig 3 shows the percentage volume deviations of the patient’s arms in the Dixon-VIBE, HUGE and MLAA AC-maps compared to the patient’s arms segmented in the NAC PET data. Patients are sorted by increasing BMI. Using the Dixon-VIBE AC-map, body volume truncations of -12.7 ± 7.1% were observed. The total range is between -3.4% up to -37.1%. Using the HUGE AC-map, truncations of -1.9 ± 3.9% were observed. The total range is between -18.6% and 6.5%. Using the MLAA AC-map body volume truncations of 7.8 ± 8.3% were observed. The total range is between -4.5% and 34.0%. All patient datasets showed truncation of the MR-based AC-maps in the region of the patients’ arms when no truncation correction was applied (Dixon-VIBE). The truncation volume in the Dixon-VIBE AC-map tends to be higher in patients with a high BMI. The truncation of patients’ arms could be reduced using HUGE, independent of the patients BMI. Note that in patient 24 one arm was missing in the HUGE AC-map due to a segmentation artifact. The segmented volumes in the HUGE AC-maps and in the NAC PET images of all other 31 patients are comparable. In patients with a lower BMI the truncation of patients’ arms could be reduced using MLAA. In patients with higher BMI, MLAA tends to overestimate the corrected arm volume when compared to the segmented NAC PET images.

**Fig 4:**
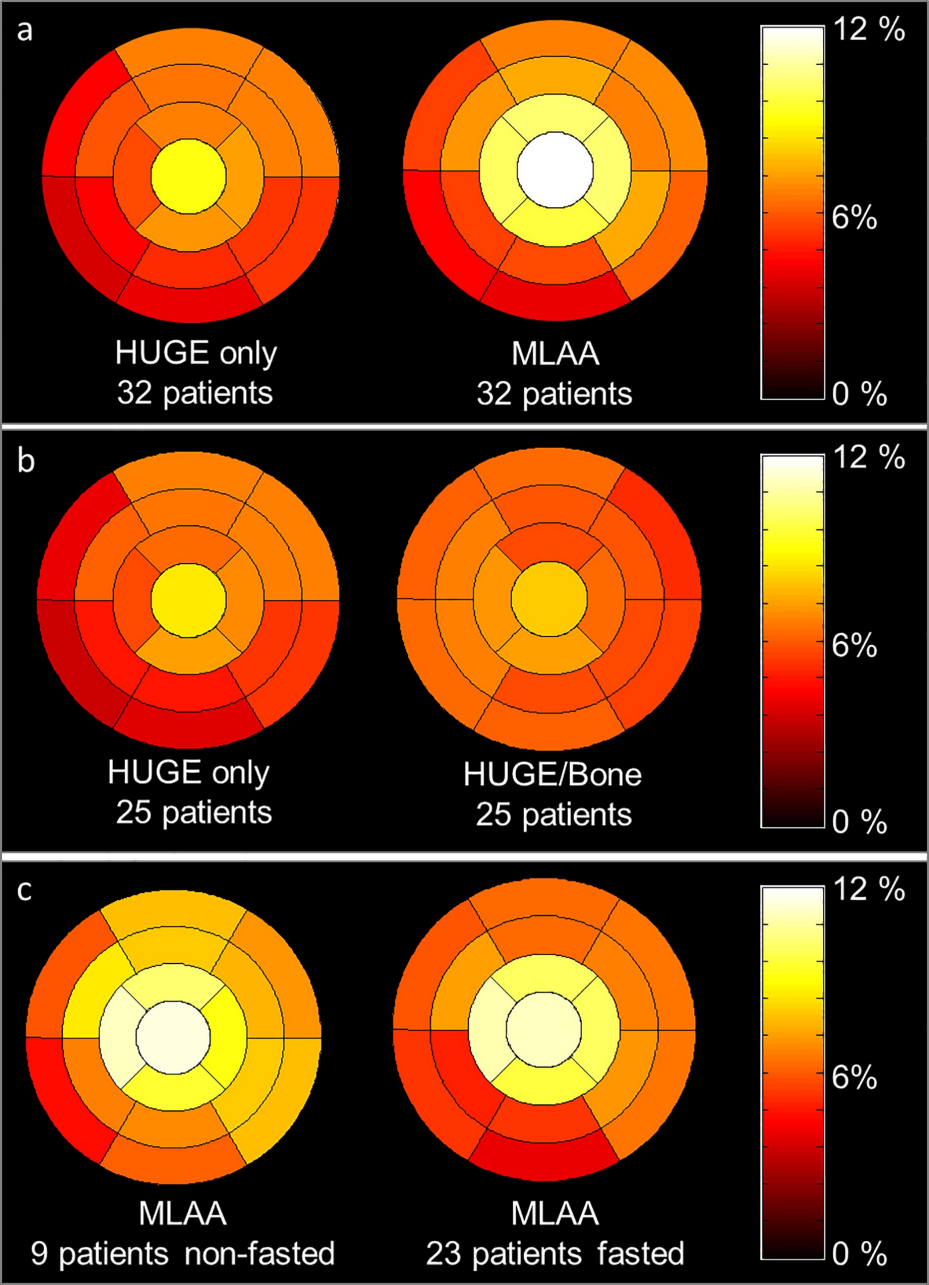
Difference polar plots between standard and improved AC. The 17-segment polar plot generated from all 32 patient PET data sets shows the average relative difference per cardiac segment between improved attenuation correction (AC) maps using HUGE truncation correction or MLAA truncation correction compared to Dixon-VIBE AC-map serving as reference (a). Polar plots in (b) show results of 25 patients after splitting the patient population into HUGE only and HUGE + bone corrected data to evaluate the effect of bone AC per segment (b). Average relative difference per segment in MLAA corrected data in patients with fasted preparation and non-fasted preparation (c) are shown to study the effect of AC on fasted and viability protocols. Note the homogeneous gain in relative difference due to HUGE and bone AC of 6% across all cardiac segments, whereas in MLAA and HUGE-only corrected data a slight decrease in relative difference inferior-medial is noticeable due to missing bone information. The relative difference over all segments and patients for MLAA is 8%. The patient preparation seems to have rather low impact on overall signal distribution in the left ventricle (c).

In Fig 4A a 17-segment polar plot is pictured which shows the average relative difference per segment of all 32 patients between truncation corrected AC-maps, respectively HUGE and MLAA, in contrast to Dixon-VIBE AC-map serving as reference standard. With regard to the comparison between HUGE only and Dixon-VIBE imaging the distribution in relative differences slightly decrease in segments 3, 4 and 9, 10. A slight increase in relative difference from base to apex from 5.9% in segments 1–6 over 6.9% in segments 13–16 and 9.5% in segment 17 is observable. With regard to the comparison between MLAA and Dixon-VIBE imaging the increase in relative difference from base to apex is also noticeable. The relative difference increases from 5.6% in segments 1–6 over 10.0% in segments 13–16 to 13.2% in segment 17. Also note the decreased relative difference inferior-medial (segments 3, 4 and 9, 10). To evaluate the effect of bone segmentation the patient population was split into HUGE only and HUGE + bone AC reconstructions (Fig 4B). In 7/32 patients the bone segmentation failed and were not corrected, therefore relative differences were analyzed just in 25/32 patients. The difference polar plot of the HUGE-only corrected data shows a decreased relative difference inferior-medial (segments 2–4 and 9, 10) comparable to the MLAA corrected data. The 25/32 patients with HUGE + bone AC show a distribution in relative difference, which is comparatively homogeneous in the left ventricle. To study the effect of improved AC on a fasted or viability protocol the patient population was split into fasted (23 patients) and non-fasted (9 patients, Fig 4C). In 6/29 patients the 18F-FDG suppression failed. Thus, the 3 true non-fasted patients and these 6 patients with failed suppression were combined in one group assuming that in end effect the failed suppression is comparable with enhanced activity. The distribution of relative differences of MLAA corrected data in the fasted and non-fasted subgroup resembles the average distribution over all patients. Therefore, the patient preparation seems to have no impact on signal distribution in the left ventricle.

The maximal relative difference in activity in almost all patients (28/32) was measured in segment 17 (apex). Nevertheless, the impact of improved AC is relatively homogeneous distributed in the polar plot of each patient. The increase in activity due to improved AC highly correlates with the increase in AC-map volume. The higher the gain in AC-map volume, the higher is the gain in activity due to improved AC.

**Fig 5:**
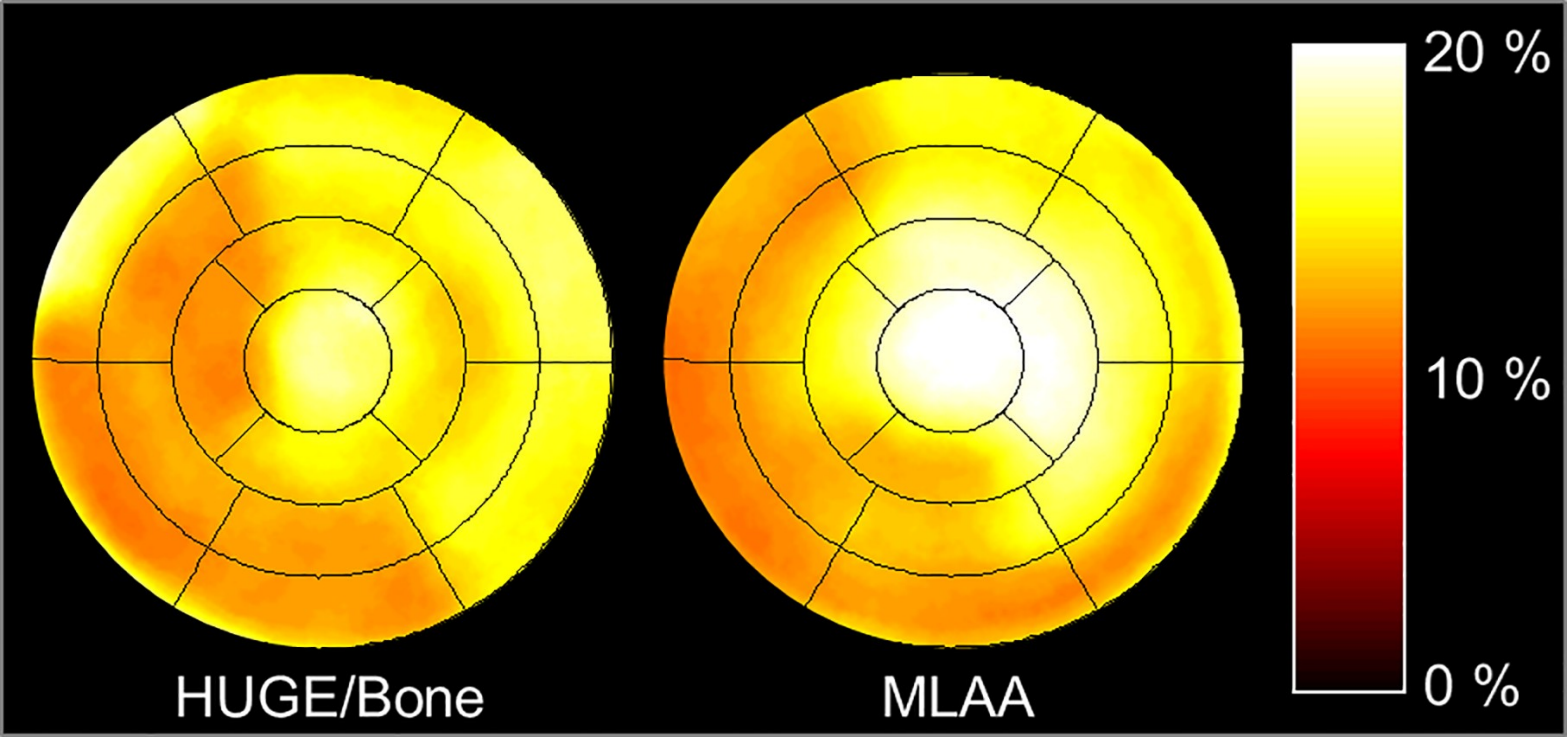
Patient example with maximal relative differences. The 17-segment polar plot of a single patient example shows the relative difference between improved attenuation correction (AC) maps using HUGE truncation correction and bone detection (a) or MLAA truncation correction (b) compared to Dixon-VIBE AC-map serving as reference. In this patient example maximal relative differences between improved AC and standard Dixon-VIBE AC were observable across the entire patient population. Note that especially in segment 17 (apex) maximal difference values with 18.8% for HUGE + bone and 19.8% for MLAA were determined.

In Fig 5 a 17-segment polar plot of a patient example shows the average relative difference per segment between improved AC-maps, respectively HUGE + bone and MLAA, in contrast to Dixon-VIBE AC-map serving as reference standard. In this example the maximal relative differences between improved AC and standard Dixon-VIBE AC were observable, especially in segment 17 (apex) with 18.8% for HUGE + bone and 19.8% for MLAA. The relative difference over all segments for HUGE + bone is 14.3% and 15.2% for MLAA compared to standard Dixon-VIBE imaging. The patient in this example has a BMI of 36.7 kg/m^2^. The gain in AC-map volume is 9.2% for HUGE and 15.7% for MLAA, the maximal values in this patient cohort.

**Fig 6:**
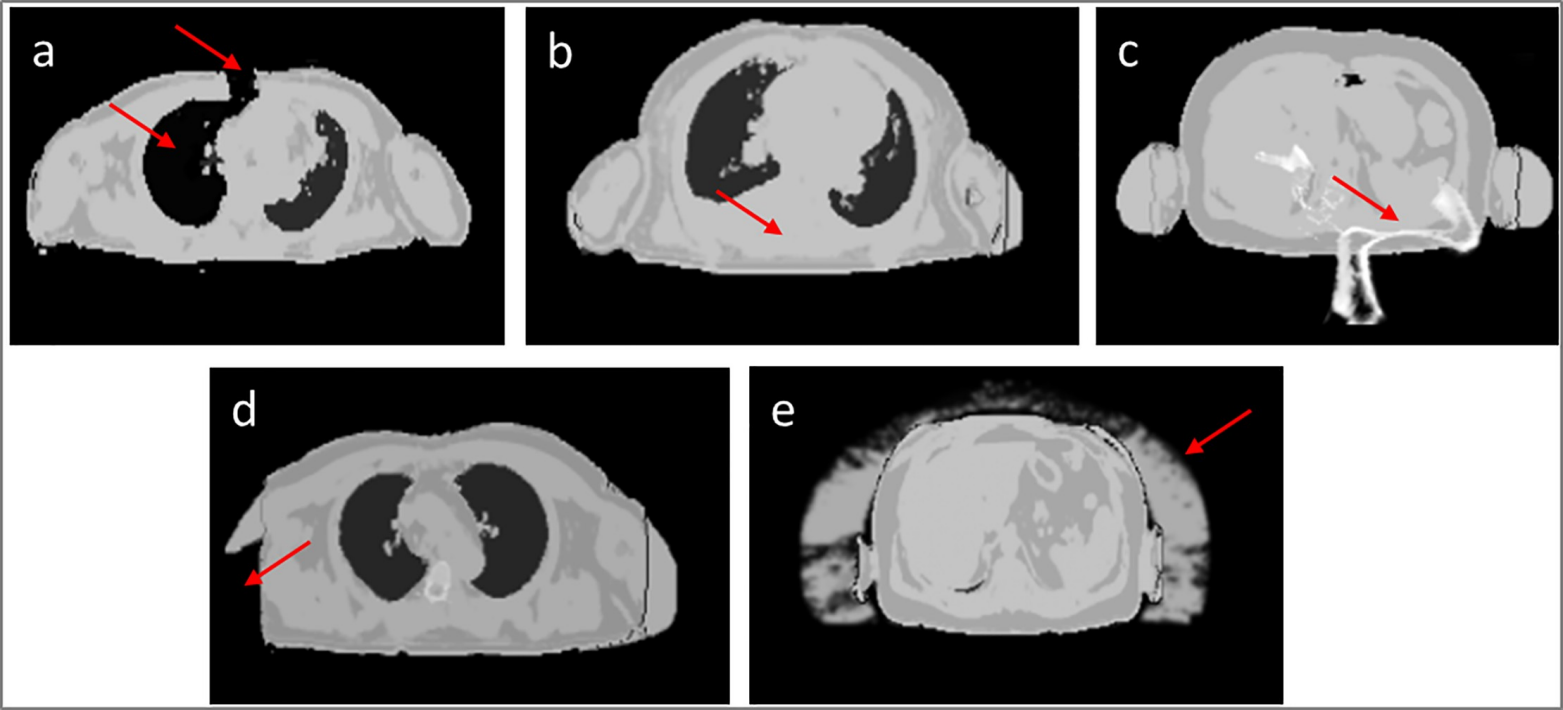
Different AC-map artifacts. Red arrows show AC-map artifacts. In 3/32 patients metal artifacts in Dixon-VIBE AC-map (caused by metal wire cerclage) leads to misclassification of surrounding tissue as “air” (a). Note that in this example the thorax is virtually opened due to the metal artifact (a). Consequently, the segmentation algorithm classifies the lung tissue of the right lung as air and assigns a lower attenuation value in this region (a). In 3/32 AC-maps no bone was segmented (b). In 4/32 patients the bone segmentation mismatches the anatomy (c). In 1/32 patient missing arm segmentation in HUGE imaging occurred (d), and in all 32/32 patients overestimation of AC-map volume in MLAA AC-maps was observed (e).

Table 1 classifies five AC-map artifacts with their origin and frequency of appearance within 32 patients. Fig 6 depicts five different AC-map artifacts that were observed in the data of 32 patients. In 3/32 patients metal artifacts in Dixon-VIBE AC-map lead to misclassification of surrounding tissue as “air” (Fig 6A). In 7/32 patients accurate bone segmentation failed (Fig 6B and 6C). Only one patient shows a HUGE-related artifact, where one arm could not be segmented (Fig 6D). In every patient an overestimation of MLAA-based AC-map volume is observable (Fig 6E).

**Table 1:** Classification of AC-map artifacts.

AC Method	Type of Artefact	Origin	Frequency
Dixon-VIBE	misclassification in Dixon-VIBE AC-map	metal artifact due to metallic implants, metallic clips or wire cerclages	3/32
Bone	missing bone segmentation	no reference for registration of bone model	3/32
Bone	mismatched bone segmentation	wrong reference for registration of bone model	4/32
HUGE	missing HUGE information	no segmentation of arms due to insufficient signal in MR sequence	1/32
MLAA	MLAA overestimation in AC-map volume	insufficient estimation of PET contour detection	32/32

Table 1 depicts the classification of five different AC-map artifacts in 32 patients.

**Fig 7:**
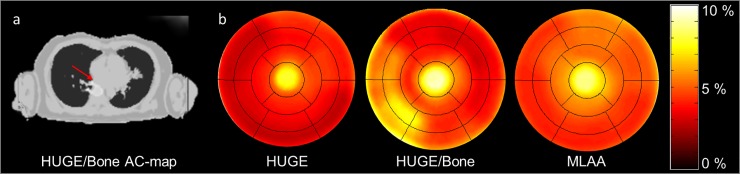
Patient example with bone artifact. Patient example with bone artefact (red arrow) in the HUGE + bone AC-map (a). Corresponding difference polar plots of HUGE only, HUGE + bone and MLAA corrected PET data in comparison to standard Dixon-VIBE AC (b). Note that HUGE only and MLAA corrected PET data show a slight decrease in relative difference inferior-medial, whereas, in HUGE and bone corrected data (middle polar plot) an increase in relative difference in segments 2–3 and 8–9 is observable.

Fig 7 shows a patient example with failed bone segmentation in the HUGE + bone AC-map. Corresponding difference polar plots of HUGE only, HUGE + bone and MLAA corrected PET data in comparison to standard Dixon-VIBE AC are shown. HUGE only corrected data display a relative difference over all segments of 4.5% and MLAA of 5.8% in contrast to standard Dixon-VIBE AC. Both approaches exhibit a decreased relative difference in inferior-medial segments 2–5 and 9–10. The HUGE + bone attenuation corrected data shows a relative difference over all segments of 5.5% in comparison to standard Dixon-VIBE AC. In segments 2–3 and 8–9 an increase of relative difference compared to Dixon-VIBE AC is observable. The maximal relative difference comparing bone (HUGE + bone) to non-bone (HUGE only) AC was observed in segment 9 with 6%.

## Discussion

The aim of this study was to evaluate and quantify the effect of improved attenuation correction including truncation correction and bone segmentation on 18F-FDG cardiac PET/MR imaging. Different methods of AC were investigated in 32 cardiac patient datasets, where the Dixon-VIBE served as the reference standard. Analysis of polar plots of the left ventricles showed that AC with HUGE + bone obtain an enhancement in activity over all patients and segments of 6.1% and MLAA of 8.3% in contrast to standard Dixon-VIBE imaging. Maximal relative differences of 18.8% for HUGE + bone and 19.8% for MLAA were observed in the apical segment.

Although no CT-based comparison was available in this study, the HUGE and MLAA AC-map were validated based on the NAC PET data of all patients serving as a reference for the non-truncated body volume. The body volume truncation of -12.7 ± 7.1% using the standard Dixon-VIBE AC method was reduced to -1.9 ± 3.9% using the HUGE approach and 7.8 ± 8.3% using the MLAA approach. According to our results in relative volume differences for HUGE and MLAA when compared against the truncated Dixon-VIBE AC-map, HUGE results in high accuracy with a slight underestimation of volume, whereas MLAA overestimates the truncated volume of almost the entire patient cohort. The systematic addition of extra volume in the AC-map due to MLAA is in agreement with the previous studies [25] [24].

All patient datasets showed truncation of the MR-based AC-maps in the region of the patients’ arms when no truncation correction was applied (Dixon-VIBE), regardless of patients’ BMI. The truncation volume in the Dixon-VIBE AC-map tends to be higher in patients with a high BMI, which is in agreement with former studies [24] [25]. The truncation of patients’ arms could be reduced using HUGE, independent of the patients BMI. In patients with a lower BMI the truncation of patients’ arms could be reduced using MLAA. In patients with higher BMI, MLAA tend to overestimate the truncated arm volume compared to the segmented NAC PET images. With regard to patients’ BMI the general effect of HUGE or MLAA compared to Dixon-VIBE imaging is intuitively larger in patients with a high BMI. A high BMI is often associated with overall large body dimensions exceeding the constraints of a conventional MR FOV. However, regarding the truncation volume and potential impact of HUGE or MLAA truncation correction on PET quantification, the overall patient dimension, and not exclusively the BMI, is the decisive factor.

The gain in AC-map volume due to FOV extension, respectively 5.4% for HUGE and 8.5% for MLAA in contrast to Dixon-VIBE AC-map (Fig 2), is in good agreement with former studies [24] [25]. When comparing the increase in global activity (HUGE 6.1% and MLAA 8.3% compared to Dixon-VIBE imaging, Fig 2) due to improved AC in the heart with the results of whole-body PET/MR studies [24] [25] [30] it is apparent that the thoracic region benefits most from enhanced AC. Adding volume of soft tissue in patients’ arms relative to the less attenuating large volume of lung tissue leads to substantial relative gains in total tissue attenuation in the thoracic region. Therefore, cardiac imaging profits from the impact of both truncation correction methods, either HUGE or MLAA.

While MR-based truncation correction using the HUGE method provides accurate contour detection, the MLAA AC-maps in almost each patient show regional overestimation in arm volume and associated artifacts from inaccurate PET image contour detection (Fig 5), which is partly compensated by applying reduced LAC values to the segmented regions. Nevertheless, the overestimation in AC-map volume results in a systematic overestimation in PET signal compared to MR-based FOV extension using HUGE (Fig 2, Fig 3). While the HUGE AC-map reconstruction worked robust and accurate for almost all patient cases, in patient #24 one arm was missing in the HUGE AC-map. In multiple attempts to reconstruct the HUGE AC-map in this specific patient example this artifact occurred. A qualitative visual inspection of the HUGE raw data depicts, that the truncation correction itself seems to work as expected. We assume that the subsequent automatic segmentation approach in the HUGE MR sequence may have failed because of insufficient signal in some regions in the MR images. This might have hampered correct segmentation and complementation of the Dixon AC map with the correctly acquired HUGE raw data in this specific patient case (S2 Fig).

The PET/MR system used in this study is not time-of-flight (TOF) capable. Therefore, the implementation of the MLAA algorithm evaluated in this study may not provide ideal results since this technique benefits from TOF capability [31] [32] [33]. Applying TOF reduces the sensitivity of PET reconstruction to AC errors and in the context of MLAA. TOF information also has the ability to stabilize the joint estimation problem [31]. To estimate the attenuation from PET data has the advantage to ensure a correct match regarding to photon energy, patient position and probably a good match in the presence of motion blurring (breathing and heart motion).

Correction of truncation artifacts using HUGE and additional bone segmentation result in a homogeneous gain in PET signal distribution of ca. 6% in the left ventricle myocardium (Fig 3). The decreased relative difference in polar plot segments 3, 4 and 9, 10 using MLAA (Fig 3) arise from missing bone information in the AC-map. The slight increase of relative difference in PET signal from base to apex may emerge from the patients’ arm posture in the PET/MR system. Due to flexing the lower arm, this region extends the MR-FOV more often, and therefore more volume is added in the AC-map here. Maximal impact of improved AC was measured in polar plot segment 17 (apex). The increased relative difference in the most apical slice may cause by partial volume effects, and therefore should be discarded from analyses.

While truncation correction of the arms with HUGE worked mostly robust, the MLAA algorithm resulted in all patient data in an overestimation of AC-map volume. No or mismatched bone segmentation may be caused by missing reference for registration of the bone model in this non-whole-body imaging application. With regard to AC-map artifacts, respectively failed bone segmentation, regional differences up to 6% within the myocardium were observed when comparing bone AC to non-bone AC and thus, may affect the clinical reading. The effect that AC-map artifacts may affect the quantitative assessment of patients was also demonstrated by Lassen et al. [9]. In their study up to 100% regional differences within the myocardium were observed due to AC-map artifacts. Readers are advised to check the AC-maps for artifacts during cardiac PET/MR image reading. Nevertheless, the majority of observed AC-map artifacts in this present work did not affect clinical reading.

While today cardiac PET/MR studies are mostly analyzed qualitatively in clinical routine, PET is well known to yield accurate quantification of global and regional myocardial perfusion and blood flow at stress and rest using PET/CT scanners [34]. Therefore, a successful implementation of myocardial PET perfusion imaging using PET/MR scanners, which is highly desirable, depends on precise MR-based AC methods [35] [36] [37] [38] [39].

A major limitation of this study is a reference for the “real” AC-map and therefore, true PET values. PET/CT cannot be used for such a reference due to several reasons: Patient positioning might be different between PET/CT and PET/MR and additionally patients were imaged arms-up in PET/CT and arms-down in PET/MR. Furthermore, pharmacokinetic factors, such as tracer kinetics, enhancement and washout over time due to different time intervals between injection and start of the PET study make an accurate quantitative comparison between the PET/MR and the PET/CT examination challenging if not impossible. A potential option to provide a ground-truth to AC is to import and register CT-based AC-maps from a PET/CT examination to retrospectively reconstruct the PET/MR data. However, most of the patients included in this study were referred to a PET/MR examination only. Just 4 patients from 32 patients first underwent a PET/CT and subsequently an additional PET/MR examination. In these cardiac PET/CT examinations the patients were imaged with arms-up, which is common in cardiac PET/CT. Therefore, a CT AC-map without arms cannot be referred to as the ground-truth or reference standard. For all these reasons, we used the MR-based standard AC-maps as reference in our study and systematically changed factors such as truncation correction and bone segmentation for a relative comparison. The inherent advantage to this method is that we could use the identical PET data set for all reconstructions, thus eliminating the impact of tracer kinetics over time.

## Conclusion

In this 18F-FDG cardiac PET/MR study the latest methods for MR-based AC, respectively the FOV extension method HUGE combined with bone segmentation, have been used and the quantitative impact on cardiac PET data have been investigated systematically. The analysis of polar plots in 32 patients has shown an overall gain in PET signal across the myocardium of the left ventricle. The results exhibited an overestimation of AC-map volume using the MLAA algorithm, while the HUGE approach resulted in a more realistic body contouring. In addition to HUGE truncation correction the incorporation of bone segmentation into the Dixon-VIBE AC-map led to a homogeneous gain in PET signal distribution across the myocardium over all examined patients. Improved attenuation correction in clinical cardiac PET/MR imaging is essential to ensure the best possible diagnostic quality. This may have clinical impact in cardiac PET/MR studies where quantitative accuracy is most important, e.g. cardiac perfusion imaging.

## Supporting information

S1 Fig HUGE raw data of two patient examples. The figure depicts the Dixon-VIBE and HUGE AC-maps, the HUGE raw data of left and right side and fusion images of the HUGE AC-map with HUGE raw data for both sides in axial and coronal orientation for 2 patient examples (#28 and #16). Note the added volume in the HUGE AC-map at patients’ arms due to truncation correction with HUGE. The HUGE method results in realistic body contouring with a slight underestimation of true arm volume.
(TIF)

S2 Fig HUGE raw data of a patient example with failed HUGE AC-map. The figure depicts the Dixon-VIBE and HUGE AC-maps, the HUGE raw data of left and right side and fusion images of the HUGE AC-map with HUGE raw data for both sides in axial and coronal orientation for patient #24 where the HUGE AC-map failed. Note that parts of the right arm are missing in the HUGE AC-map due to failed segmentation in some regions (red arrows). HUGE raw data show the truncation correction in those regions (red arrows). In this single case the HUGE AC-map segmentation failed despite correct acquisition of HUGE raw data.
(TIF)

S1 Table Detailed patient information. The table lists all relevant patient data, e.g. body mass index (BMI) and the post injection time (p.i.). Note that patient numbering in Fig 3 does not correspond with this list as patients in Fig 3 are sorted by increasing BMI.
(TIF)

S2 Table Total segmented volume of AC-maps. The table lists the segmented total volume of AC-maps (reference) and calculated relative differences of the HUGE and MLAA-based AC-maps compared to Dixon AC-maps. Note that patient numbering in Fig 3 does not correspond with this list as patients in Fig 3 are sorted by increasing BMI.
(TIF)

S3 Table Activity in polar plot segments. The table lists the activity in each polar plot segment and calculated relative differences of the HUGE and MLAA corrected PET images compared to Dixon-VIBE corrected PET images. Note that patient numbering in Fig 3 does not correspond with this list as patients in Fig 3 are sorted by increasing BMI.
(XLSX)
