# Supplementary figures and images for: Impact of improved attenuation correction on 18F-FDG PET/MR hybrid imaging of the heart
Source: PLoS One. 2019 Mar 25;14(3):e0214095. doi: 10.1371/journal.pone.0214095 (PMC6433217; doi:10.1371/journal.pone.0214095)

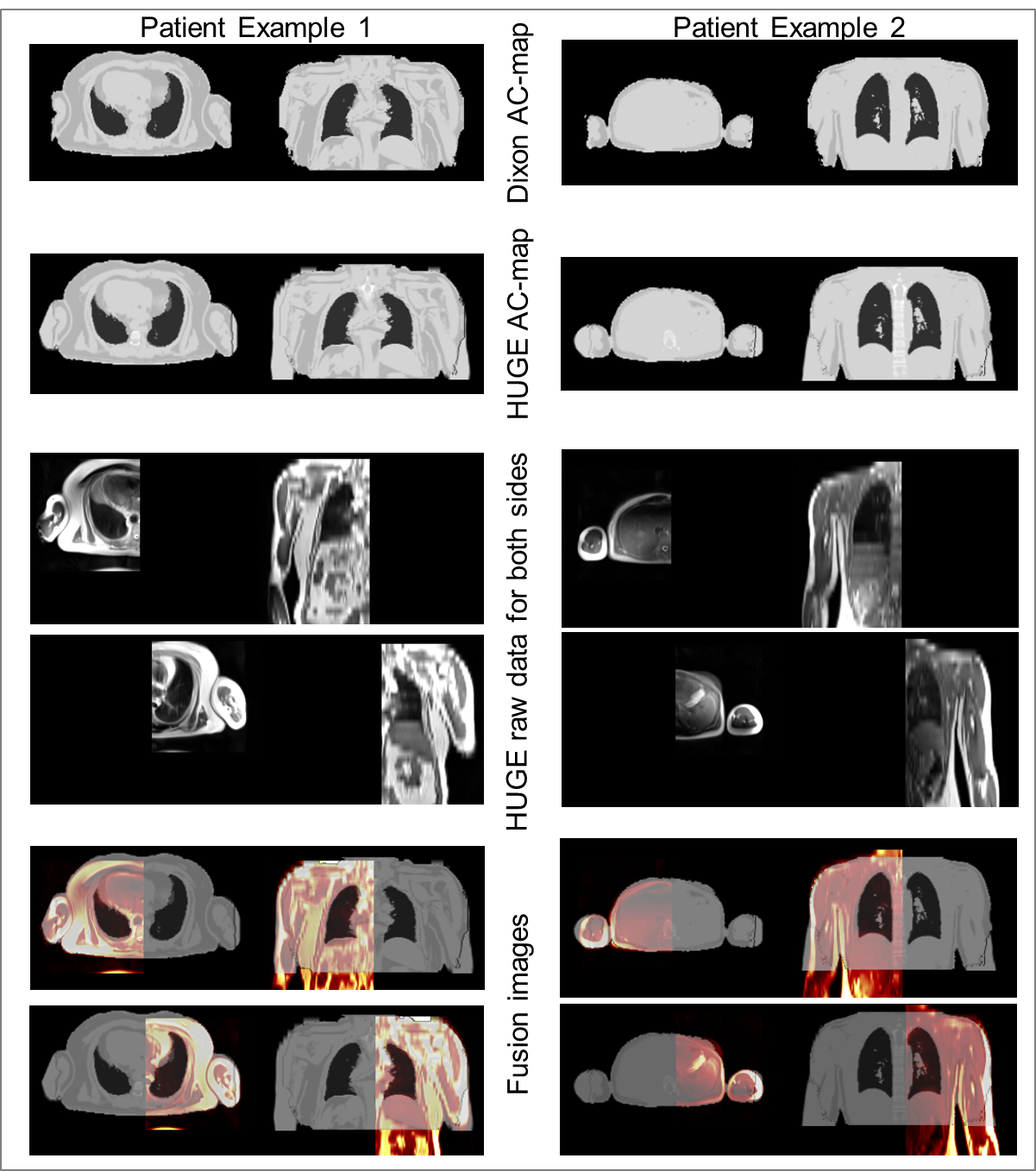

Supplement: S1 Fig — The figure depicts the Dixon-VIBE and HUGE AC-maps, the HUGE raw data of left and right side and fusion images of the HUGE AC-map with HUGE raw data for both sides in axial and coronal orientation for 2 patient examples (#28 and #16). Note the added volume in the HUGE AC-map at patients’ arms due to truncation correction with HUGE. The HUGE method results in realistic body contouring with a slight underestimation of true arm volume. (TIF) [file pone.0214095.s001.tif]

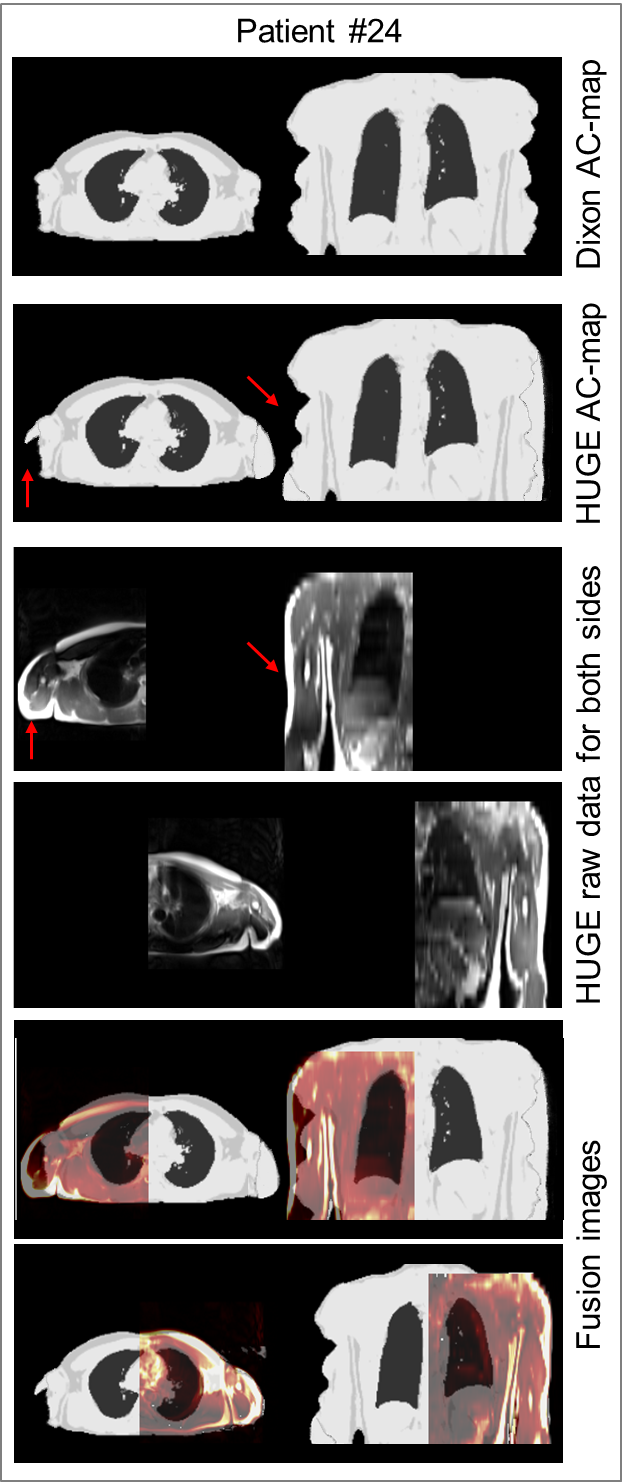

Supplement: S2 Fig — The figure depicts the Dixon-VIBE and HUGE AC-maps, the HUGE raw data of left and right side and fusion images of the HUGE AC-map with HUGE raw data for both sides in axial and coronal orientation for patient #24 where the HUGE AC-map failed. Note that parts of the right arm are missing in the HUGE AC-map due to failed segmentation in some regions (red arrows). HUGE raw data show the truncation correction in those regions (red arrows). In this single case the HUGE AC-map segmentation failed despite correct acquisition of HUGE raw data. (TIF) [file pone.0214095.s002.tif]

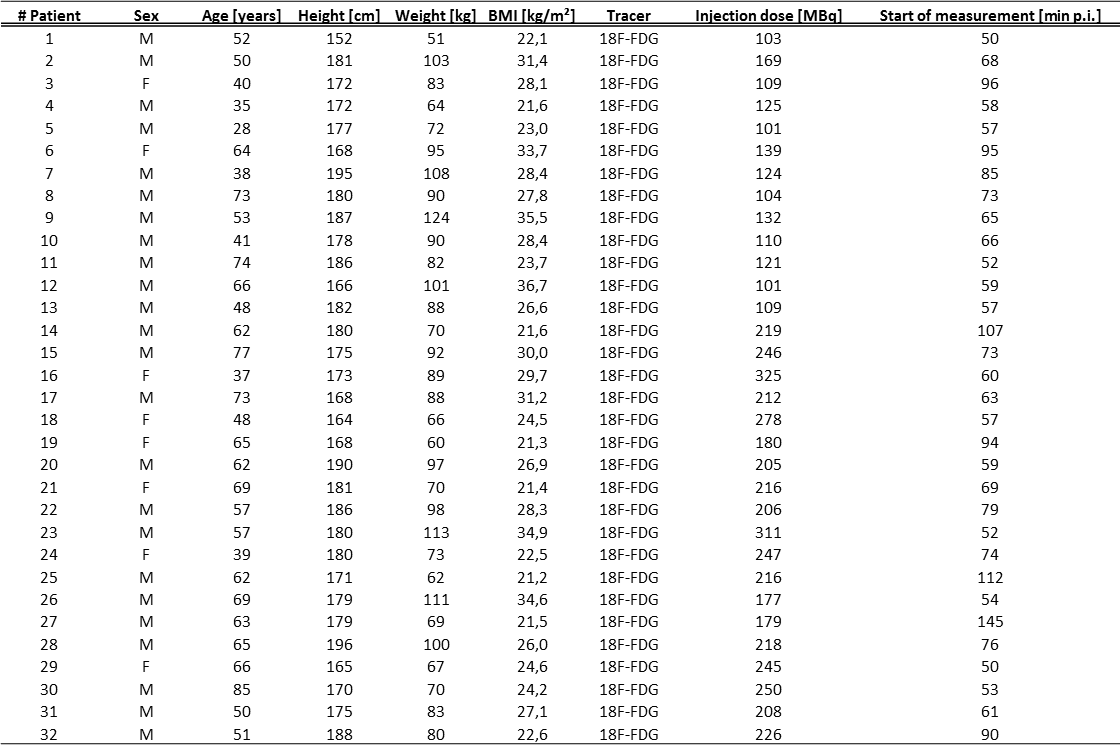

Supplement: S1 Table — The table lists all relevant patient data, e.g. body mass index (BMI) and the post injection time (p.i.). Note that patient numbering in Fig 3 does not correspond with this list as patients in Fig 3 are sorted by increasing BMI. (TIF) [file pone.0214095.s003.tif]

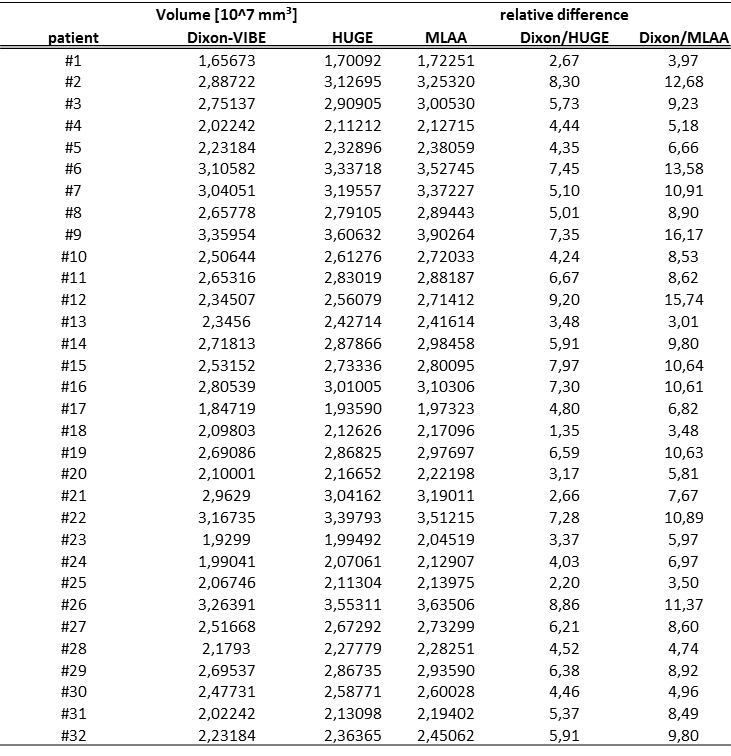

Supplement: S2 Table — The table lists the segmented total volume of AC-maps (reference) and calculated relative differences of the HUGE and MLAA-based AC-maps compared to Dixon AC-maps. Note that patient numbering in Fig 3 does not correspond with this list as patients in Fig 3 are sorted by increasing BMI. (TIF) [file pone.0214095.s004.tif]
